# Working Memory During Late Pregnancy: Associations With Antepartum and Postpartum Depression Symptoms

**DOI:** 10.3389/fgwh.2022.820353

**Published:** 2022-02-23

**Authors:** Iliana Liakea, Ashish K. C., Emma Bränn, Emma Fransson, Inger Sundström Poromaa, Fotios C. Papadopoulos, Alkistis Skalkidou

**Affiliations:** ^1^Department of Women's and Children's Health, Uppsala University, Uppsala, Sweden; ^2^Department of Medical Sciences, Psychiatry, Uppsala University, Uppsala, Sweden

**Keywords:** working memory, short-term memory/attention, postpartum depression, antepartum depression, Edinburgh Postnatal Depression Scale

## Abstract

**Background:**

Few studies, with conflicting results, report on the association between memory performance and depressive symptoms during the perinatal period. In this study, we aimed to evaluate whether memory performance during late pregnancy is associated with antepartum (APD) and postpartum depression (PPD) symptoms.

**Method:**

We conducted a prospective follow-up of 283 pregnant women, nested within a large cohort of women enrolled in the BASIC study in Uppsala University hospital between 2009 and 2019. The Wechsler Digit Span Task (forward-DSF, backward-DSB and total score-DST) was performed to evaluate short-term memory/attention (DSF) and working memory (DSB) around the 38th gestational week; the Edinburgh Postnatal Depression Scale (EPDS), evaluating depressive symptoms, was filled out at 17, 32, 38 gestational weeks, as well as at 6 weeks postpartum. Unadjusted and multivariate logistic regression was used to assess the association between performance on the Digit Span Task and outcome, namely depressive symptoms (using a cut-off of 12 points on the EPDS) at 38 gestational weeks, as well as at 6 weeks postpartum.

**Results:**

APD symptoms were not significantly associated with DSF (*p* = 0.769) or DSB (*p* = 0.360). APD symptoms were significantly associated with PPD symptoms (*p* < 0.001). Unadjusted regression modeling showed that DSF in pregnancy was a significant predictor of PPD symptoms (OR 1.15; 95% CI, 1.00, 1.33, *p* = 0.049), and remained a significant predictor when adjusted for confounders (education and feeling rested at assessment; OR 1.21, 95% CI 1.03, 1.42, *p* = 0.022). DSF was a predictor of PPD symptoms only for women without a pre-pregnancy history of depression (OR 1.32; 95% CI 1.04, 1.67, *p* = 0.024) and also those without APD (OR 1.20, 95% CI 1.01, 1.43, *p* = 0.040).

**Conclusion:**

There was no significant association between working and short-term memory performance and APD symptoms. Among all women, but especially non-depressed earlier in life and/or at antepartum, those scoring high on the forward memory test, i.e., short-term memory, had a higher risk for PPD. Future studies are required to further explore the pathophysiology behind and the predictive value of these associations.

## Introduction

Pregnancy and the postpartum period constitute one of the most vulnerable periods of women's life, wherein women undergo surmountable hormonal, mental and physiological changes. Short-term cognitive impairment, popularly named as “pregnancy/baby brain” in terms of increased forgetfulness, short attention span and disorientation has been widely reported (1–5). This “baby brain” phenomenon is widely encountered, since up to 80% of pregnant women report such subjective signs of cognitive impairments (6).

Current research on short-term cognitive impairments during pregnancy also employs objective neuropsychological measures to pinpoint them (7). Contradictory results have complicated the understanding of cognition during pregnancy (4, 7, 8). Some studies report no effect of pregnancy on cognition (9, 10) and others present evidence of cognitive deficiencies in specific domains (11, 12). Meta-analyses on the short-term cognitive impairments during pregnancy report a modest reduction in verbal recall memory, working memory, and prospective memory (4, 7, 8). Interestingly, based on the latest meta-analysis (7), short-term cognitive impairments need to also be studied in the context of depressive symptoms during pregnancy.

The prevalence of depression during pregnancy, known as antepartum depression (APD), is ~15% according to the estimate of a recent systematic review (13). This is a rather noteworthy prevalence, since APD has been linked with a range of adverse outcomes, including preeclampsia, low birth weight, preterm delivery, and both cognitive and behavioral deficits in the child (14). Also, the APD symptoms are comparable to major depressive disorder's (MDD) symptomatology. In fact, APD is considered a unipolar, non-psychotic depressive episode that begins in or extends into pregnancy (15, 16). Like depressive episodes, APD has been found to be accompanied by cognitive impairments (7, 17).

However, literature presents a controversy regarding the timeline of depression onset and the manifestation of cognitive impairments. It has been suggested that cognitive impairments may precede depression onset, co-appear, or even exist when depression is in remission (18). It has been suggested that low cognitive ability in early adulthood is a risk factor for later development of depressive symptomatology and a possible explanation of that was that less cognitive capable individuals when experience elevated levels of stress adopt less flexible coping strategies and have fewer resources to allocate (19). Adjusting this model to the period of pregnancy, when the transition to motherhood can be considered as a significant life event, and adjustment in new relationships, roles, and routines are needed to be made (20), it can be hypothesized that fewer available cognitive resources and less flexible coping strategies may complicate the transition and trigger depressive symptoms. Depressive symptoms that arise after labor may be signs of postpartum depression (PPD). PPD is a major depressive episode with onset in the postpartum period, with an estimated global prevalence of 17.2% (21). In Sweden, the estimated prevalence is 12.2% (21) and it ranges between 8 (22) and 14.6% (23) of all mothers giving birth. Also, the symptoms of PPD last more than 6 months for 25–50% of those affected (24). If PPD is not identified and properly treated, it can have long-lasting effects on both the mother and the child and their relationship. PPD is related to an elevated risk of delay in cognitive, language and motor milestones (25, 26).

The prevalence and the adverse effects of APD and PPD create a need for researching possible predictors and intervening on them. To our knowledge, current research has partially established the association between short-term cognitive impairments with APD but has not examined a similar relationship with PPD. In fact, it has been shown that pregnant women with a high or moderate level of depressive and/or anxiety symptoms performed significantly worse in visuospatial working memory/executive functioning tasks than women with low symptom levels (27). In addition, Hampson et al. (28) have found that pregnant women with depressive symptomatology exhibited poorer working memory performance compared to non-pregnant controls and pregnant women without depressive symptomatology. However, there are studies that do not confirm this pattern. Christensen and his colleagues (1) explored whether pregnancy is associated with cognitive impairments, such as working memory and immediate recall. To control for confounding variables, they examined the associations between several identified confounding variables, among which was depression during pregnancy, and each cognitive outcome. However, APD was not significantly related to any measured cognitive impairment.

Apparently, the findings on whether working memory impairments during pregnancy are associated with APD are contradictory. Working memory refers to the ability to maintain and/or manipulate information within the memory store (29). Several studies that investigated pregnant women's performance on working memory have used the Digit Span Backward task [DSB; (7)] which is part of the WAIS-III Digit Span test (30). The Digit Span task entails, beyond the DSB, the Digit Span Forward task (DSF) which measures attention, encoding, and auditory processing (31, 32). This measurement has been used to assess attention and short-term memory (5, 12) since it requires participants' attention to the order of the digits and immediate memory to store the information temporarily. DSB is measuring working memory, mental manipulation, visuospatial imaging, and transformation of information since participants need to repeat the digits they have heard in the reverse order (33, 34). Congruently, Rudel and Denckla (35) believed that the backward task requires the transformation of the sequential order into left-right spatial coordinates. Recent evidence has pinpointed the dorsolateral prefrontal cortex (DLPFC) as a brain area related to working memory (29, 36).

Given the possible importance of short-term cognitive impairments during pregnancy and their potential predictive value in detecting the development of PPD, the current study has 2-fold objectives. First, to assess whether pregnant women with depressive symptoms face working memory impairments. Second, to assess whether impairments in working and/or short-term memory can be predictors of PPD development, especially clinically relevant among healthy pregnant women. We took into consideration the possible impacts of the “scar effect,” according to which cognitive impairments can be present even when MDD is in remission (37); we therefore also stratified analyses by history of depression.

## Materials and Methods

### Participants

This study is a nested study within a large longitudinal population-based cohort project at the Uppsala University Hospital. Twenty-one percent (*N* = 6,478) of the pregnant women attending the routine ultrasound examination at the Uppsala University Hospital at gestational weeks 16–18 agreed to participate in the BASIC (Biology, Affect, Stress, Imaging, and Cognition) study. To be eligible for the study, women needed to meet the following inclusion criteria (a) to be over 18 years old, (b) to communicate adequately in Swedish, (c) to have a normal pregnancy as diagnosed by routine ultrasound, (d) not having confidential personal data, and (e) not having any known blood-borne disease. More information about the study is provided elsewhere (38). The study was conducted between 2009 and 2018.

In this study, we included women from the BASIC who participated in a more thorough assessment at the women's research laboratory (*n* = 340) around gestational week 38. For recruiting this subgroup of participants, invitations were sent to women scoring 12 or more on the Edinburgh Postnatal Depression Scale (EPDS) in gestational week 32, to oversample cases with ongoing depression. Nearly as many women were invited as controls, i.e., those scoring 0–11 on the EPDS. To assess if subgroup's sample characteristics substantially differ from the BASIC cohort sample, we compared their background, pregnancy and postpartum related characteristics. Only the prevalence of history of depression and APD symptoms were higher in our sample compared to the remaining BASIC cohort, which is expected based on the selection of the subgroup.

We excluded women who received selective serotonin reuptake inhibitors (SSRI) medication (*n* = 57). We decided to employ this exclusion criterion because SSRIs in themselves have been found to improve memory performance (39). Therefore, the sample consisted of *N* = 283 women, 246 out of which did not manifest significant depressive symptoms at gestational week 38 when the memory performance task took place.

### Assessments

The Wechsler Digit Span Task (30) is a measurement of working memory and attention. In particular, DSF measures attention, concentration, mental control, and coding, while DSB measure mental speed, and working memory (30). The examiner verbally presents digits to the participant at a rate of 1 per second in a specific order. Participants at the DSF task are instructed to repeat the digits verbatim, whereas at the DSB task they are asked to repeat the digits in reverse order. The test starts with a trial of 2 digits, subsequently increasing by one until 9 (DSF) and 8 digits (DSB) are achieved. Each trial was administered twice, using different numbers, even if the women got Trial 1 correctly. For each trial, 1 point is scored for a correct response or 0 points for an incorrect response or no response. DSF's scores range between 0 and 16 whereas DSB's scores range between 0 and 14. When participants fail two trials in a row, the task finishes. The completed trials for DSF and DSB are calculated separately giving a summarized score for DSF and for DSB. The score from DSF and DSB is further summarized into a total score (DST). This task was performed by participants during a visit to the research laboratory during late pregnancy (35–39 weeks).

The Edinburgh Postnatal Depression Scale (EPDS) (40) is a 10-item self-report screening tool including questions regarding common depressive symptoms, but excluding somatic symptoms, such as fatigue and change in appetite that may be expected during pregnancy. Each item is scored on a 4-point Likert scale ranging between 0 and 3, with higher scores indicating greater depressive symptomatology. A Swedish validation study has shown that for the optimal cut-off score of 12 or more, the EPDS yields high sensitivity and specificity (41). We assessed women with the EPDS at the 17th, 32nd, and 38th gestational week and the 6th week postpartum. To define antepartum depression, we considered only the outcome of the assessment at the 38th gestational week, as memory performance was assessed at the same time point, and dichotomized women in those having 12 or more points in the EPDS or less. Analyses were nevertheless performed even with depression status earlier in pregnancy, in case there would be a lag effect of depression. Similarly, women at 6 weeks postpartum were dichotomized in those scoring above 12 in the EPDS or less.

**Covariates** were selected based on relevant literature regarding working memory as well as perinatal depression. Covariates included in the adjusted analysis were maternal education, assessed as a dichotomous variable (university/college education vs. others), and feeling rested at assessment, assessed as a categorical variable (yes vs. no).

### Statistical Analyses

**Figure 1:**
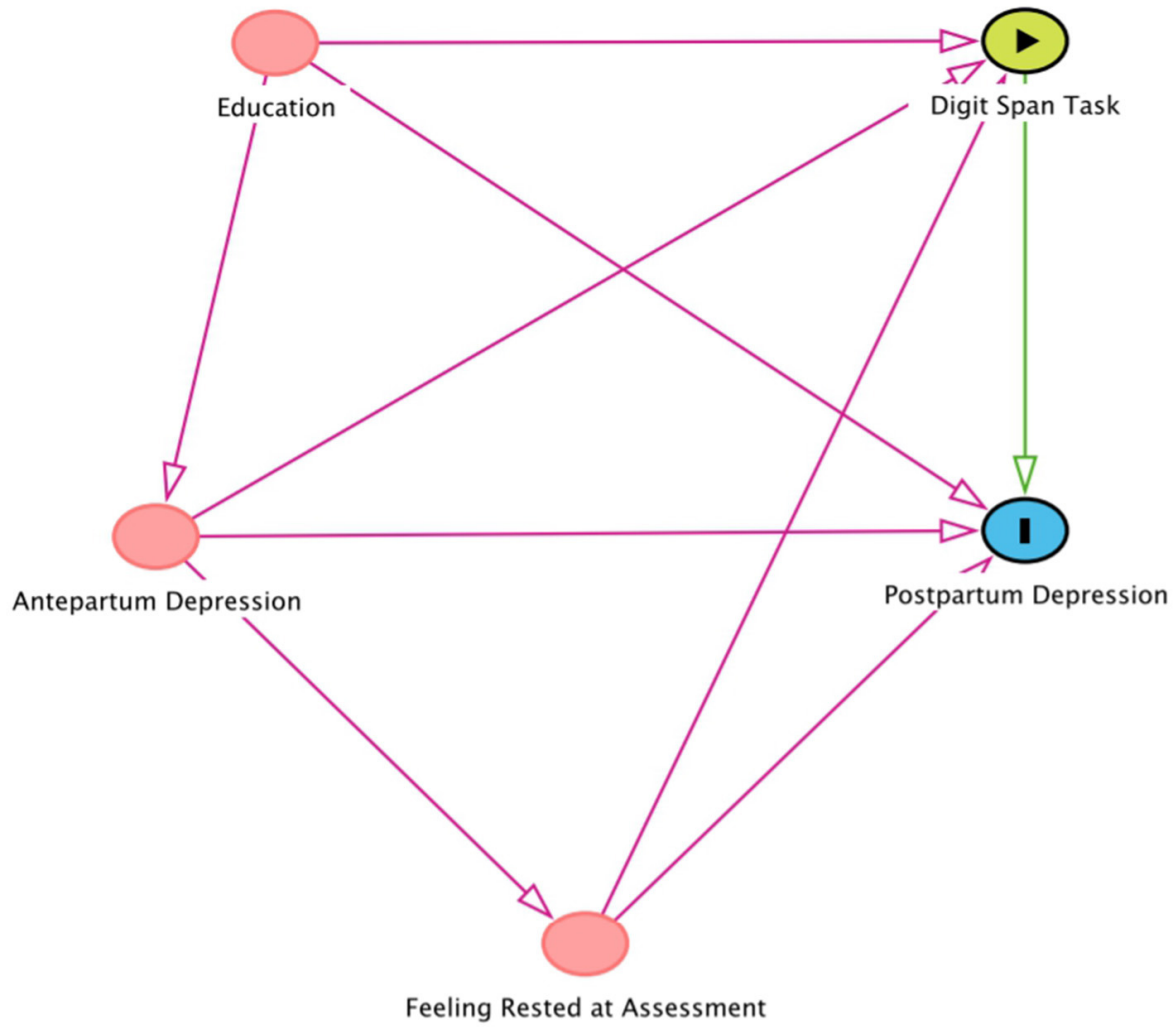
The Figure represents the Direct Acyclical Graph (DAG) depicting hypothesized associations between the main study variables, pertaining mostly to Aim 2. Antepartum Depression, education and feeling rested at assessment, according to DAG system, are the variables of interest in the association of Digit Span Task with Postpartum Depression. According to the DAG model, we have adjusted for education and feeling rested at assessment, and stratified for Antepartum Depression.

Descriptive statistics for continuous variables were presented using mean and standard deviation (mean ± sd), while for the categorical variables the absolute and relative frequencies (*n*, %) were calculated. Unadjusted associations of the memory test scores vs. background characteristics and several factors of interest during pregnancy and postpartum were assessed using Spearman Rho correlations and Student's *t*-test where appropriate. Among those variables of interest is APD. Likewise, the unadjusted associations of depressive symptomatology at 6 weeks postpartum (EPDS total score ≥12), were assessed by performing Student's *t*-test or Pearson's chi-square test where appropriate. In order to assess the adjusted associations between the memory test during pregnancy and depressive symptoms at 6 weeks postpartum, 2 nested logistic regression models were estimated. In the first model, the unadjusted associations between the memory test during pregnancy and depressive symptoms postpartum were estimated. In the second model the above associations were adjusted for education and feeling rested at assessment. The models' goodness of fit was evaluated through the Hosmer-Lemeshow statistic. Additionally, Spearman correlations between the memory test and the total EPDS score at 6 weeks postpartum were calculated and the corresponding scatterplots were constructed. All of the above analyses were repeated after stratifying for history of depression and depression status at the 38th gestational week. Figure 1 presents a Direct Acyclical Graph (DAG), depicting hypothesized associations between the main study variables. Education and feeling rested at assessment were treated as confounders, while for Aim 2, we stratified by APD, as per the study aim. All reported *p*-values were based on two-sided tests and the 5% significance level was used for hypothesis testing. Analyses were performed using the SPSS software, version 26.0 and the figures were constructed using the STATA 14 software.

## Results

The descriptive statistics of the study population are presented in Table 1. Most participants had a higher degree education (79.0%) and more than 50.0% of women were multiparas. Also, most of the women did not have a history of depression (60.3%), neither had depressive symptomatology during pregnancy (86.9%).

**Table 1:** Background, pregnancy and postpartum related characteristics of the study population (*n* = 283).

		***n***	**Mean ± SD**	***%***
Maternal age at partus		283	31.40 ± 4.31	
BMI before pregnancy		282	23.65 ± 4.01	
Feeling rested at assessment	No	116		41.7
	Yes	162		58.3
Parity	Primipara	134		47.7
	Multipara	147		52.3
Education	University	222		79.0
	Else	59		21.0
Pre-pregnancy history of depression	No	167		60.3
	Yes	110		39.7
Antepartum depression	No	246		86.9
	Yes	37		13.1
Worried about delivery	No	220		78.6
	Yes	60		21.4
Delivery experience	Positive	224		90.3
	Negative	24		9.7
Sleep (6 weeks postpartum)	>6 h	119		42.3
	≤6 h	162		57.7
Partner helps with babycare (6 weeks postpartum)	No help/Some help	101		36.2
	Much help	178		63.8

### Maternal Background Characteristics in Relation to Memory Performance

Women with higher/university education performed significantly better than their counterparts on memory tasks (*p* < 0.001). No other bivariate associations were noted (see Table 2).

**Table 2:** Background characteristics in relation to performance on Digit Span Task (*n* = 283).

		**DSF**			**DSB**			**DST**		
		***n***	**Mean ± SD**	***P***	***n***	**Mean ± SD**	***p***	***n***	**Mean ± SD**	***P***
			***Spearman rho***			***Spearman rho***			***Spearman rho***	
Maternal age at partus		283	*−0.03*	0.636	282	*0.01*	0.869	281	*−0.02*	0.761
BMI before pregnancy		282	*0.00*	0.982	281	*0.03*	0.581	280	*0.02*	0.716
Feeling rested at assessment	No	116	9.5 ± 2.26	0.344	116	6.8 ± 2.16	0.457	115	16.2 ± 3.81	0.405
	Yes	162	9.3 ± 2.13		161	6.6 ± 1.97		161	15.8 ± 3.69	
Parity	Primipara	134	9.5 ± 2.29	0.281	133	6.7 ± 2.17	0.707	133	16.1 ± 3.99	0.368
	Multipara	147	9.2 ± 2.06		147	6.6 ± 1.94		146	15.7 ± 3.48	
Education	Else	59	8.4 ± 1.68	**<0.001**	58	5.8 ± 1.90	**<0.001**	58	14.1 ± 3.14	**<0.001**
	University	222	9.6 ± 2.25		222	6.9 ± 2.02		221	16.4 ± 3.74	
Antepartum depression	No	246	9.4 ± 2.19	0.769	245	6.7 ± 2.06	0.360	244	16.0 ± 3.75	0.789
	Yes	37	9.5 ± 2.32		37	6.4 ± 1.89		37	15.9 ± 3.73	
Worried about delivery	No	220	9.4 ± 2.21	0.940	219	6.7 ± 2.11	0.750	219	16.0 ± 3.86	0.746
	Yes	60	9.4 ± 2.16		60	6.6 ± 1.80		59	15.8 ± 3.32	

*DSF, Digit Span Forward; DSB, Digit Span Backwards; DST, Digit Span Total which is the sum of DSF and DSB. Spearman correlation analyses were performed between maternal age and DSF/DSB/DST and BMI before pregnancy and DSF/DSB/DST. Student's t-test analyses were performed for all categorical variables. Values of the Spearman rho are reported in italics. P values of significant associations and differences are reported in bold*.

### Associations Between Memory Performance and Depressive Symptoms at 38 Gestational Weeks

Women with APD symptoms did not perform significantly differently on memory tasks than those without depressive symptoms during pregnancy (see Table 2). This pattern of results remained the same even when we tested associations between memory performance and early and mid-pregnancy depression instead.

### Maternal Background Characteristics in Relation to Depressive Symptoms at 6 Weeks Postpartum

Women who had greater depressive symptoms at 6 weeks postpartum performed better on DSF than those who scored lower on EPDS (*p* = 0.047). No other memory-related outcome was associated with EPDS. Also, women with higher scores on depressive symptoms at 6 weeks postpartum had higher BMI before pregnancy compared to those with lower EPDS scores (*p* = 0.029). EPDS at 6 weeks postpartum was related with feeling rested at assessment (*p* = 0.001), pre-pregnancy history of depression (*p* < 0.001), antepartum depression (*p* < 0.001), being worried about delivery (*p* = 0.012), delivery experience (*p* = 0.023), sleep at 6 weeks postpartum (*p* = 0.020) and partner's support (*p* = 0.006) (see Table 3). No other significant associations were identified.

**Table 3:** Background characteristics in relation to depressive symptoms measured by Edinburgh Postnatal Depression Scale (EPDS) at 6 weeks postpartum (*n* = 283).

		**Depressive symptoms 6 weeks postpartum**				***P*** **-value**
		**EPDS 0–11 (*****n*** **= 238)**		**EPDS 12–30 (*****n*** **= 45)**		
		***n***	***Mean ± SD*** **%**	***n***	***Mean ± SD*** **%**	
Age at partus (years)		152	*32 ± 4.3*	45	*31 ± 4.1*	0.203
BMI before pregnancy (kg/m^2^)		237	*23 ± 3.8*	45	*25 ± 4.8*	**0.029**
Feeling rested at assessment	No	89	38%	27	64%	**0.001**
	Yes	147	62%	15	36%	
Parity	Primipara	113	48%	21	48%	0.995
	Multipara	124	52%	23	52%	
Education	Else	46	19%	13	29%	0.156
	University	190	81%	32	71%	
Pre-pregnancy history of depression	No	152	66%	15	33%	**<0.001**
	Yes	80	34%	30	67%	
Antepartum depression	No	217	92%	29	64%	**<0.001**
	Yes	21	8%	16	36%	
Worried about delivery	No	191	81%	29	64%	**0.012**
	Yes	44	19%	16	36%	
Fear of childbirth	No fear	180	76%	28	64%	0.087
	Any fear	57	24%	16	36%	
Delivery experience	Positive	192	92%	32	80%	**0.023**
	Negative	16	8%	8	20%	
Sleep (6 weeks postpartum)	More than 6 h	107	45%	12	27%	**0.020**
	6 h or less	129	55%	33	73%	
Partner helps with baby care (6 weeks postpartum)	No help/Some help	77	33%	24	55%	**0.006**
	Yes much help	158	67%	20	45%	
DSF		238	*9.2 ± 2.1*	45	*10 ± 2.6*	**0.047**
DSB		237	*6.6 ± 2.0*	45	*6.7 ± 2.2*	0.856
DST		236	*16 ± 3.6*	45	*17 ± 4.2*	0.184

*EPDS, Edinburgh Postnatal Depression Scale; DSF, Digit Span Forward; DSB, Digit Span Backward; DST, Digit Span Total which is the sum of DSF and DSB. Chi-square for independence was performed between categories of EPDS at 6 week postpartum and categorical variables. Values of the Spearman rho are reported in italics. P values of significant associations and differences are reported in bold*.

### Associations Between Memory Performance and Depressive Symptoms at 6 Weeks Postpartum

To assess the predictive role of memory performance in late pregnancy on depressive symptoms at 6 weeks postpartum, we run logistic regressions (see Table 4). In the unadjusted regression analysis, DSF positively predicted scoring above the cut-off on the EPDS at 6 weeks postpartum (OR 1.15, 95% CI 1.00, 1.33, *p* = 0.049). In the adjusted models, again only DSF significantly predicted EPDS score at 6 weeks postpartum (OR 1.21, 95% CI 1.03, 1.42, *p* = 0.022).

**Table 4:** Logistic regression models evaluating the unadjusted and adjusted associations of the performance on Digit Span Task (predictor) with depression status at 6 weeks postpartum (outcome) (*n* = 283).

	**DSF**		**DSB**		**DST**	
	**OR (95% CI)**	***P*** **-value**	**OR (95% CI)**	***P*** **-value**	**OR (95% CI)**	***P*** **-value**
Model 1^a^	1.15 (1.00, 1.33)	**0.049**	1.02 (0.87, 1.19)	0.856	1.06 (0.97, 1.15)	0.184
Model 2^b^	1.21 (1.03, 1.42)	**0.022**	1.06 (0.90, 1.25)	0.467	1.09 (0.99, 1.20)	0.065

a *Unadjusted associations between the performance on the Digit Span Task and depression status at 6 weeks postpartum, based on an cut-off of 12 points on the Edinburgh Postnatal Depression Scale (EPDS)*.

b *Adjusted associations for: education and feeling rested at assessment. DSF, Digit Span Forward; DSB, Digit Span Backward; DST, Digit Span Total which is the sum of DSF and DSB. P values of significant associations are reported in bold*.

Spearman correlations between memory performance score and total EPDS score at 6 weeks postpartum were not statistically significant.

### Associations Between Memory Performance and Depressive Symptoms at 6 Weeks Postpartum After Stratifying by the Pre-pregnancy History of Depression

To account for a potential “scar effect” of a history of depression, we run the models after stratifying for this variable. For women without history of depression, DSF (OR 1.32 95% CI 1.04, 1.67, *p* = 0.024) and DST (OR 1.14 95% CI 1.00, 1.30, *p* = 0.045) predicted depressive symptoms at 6 weeks postpartum. However, when adjusted for education and feeling rested at assessment, DSF and DST did not remain predictors of EPDS score at 6 weeks postpartum. For women with a history of depression, memory performance was not a predictor of EPDS score at 6 weeks postpartum (see Supplementary Table 1).

### Associations Between Memory Performance and Depressive Symptoms at 6 Weeks Postpartum After Stratifying by EPDS Score at 38 Gestational Weeks

The stratified unadjusted logistic regression revealed that DSF was positively associated with EPDS at 6 weeks postpartum only among women not presenting depressive symptomatology in late pregnancy (OR 1.20, 95% CI 1.01, 1.43, *p* = 0.040). This association remained significant in the adjusted model (OR 1.25, 95% CI 1.03, 1.51, *p* = 0.024; see Supplementary Table 2).

**Figure 2:**
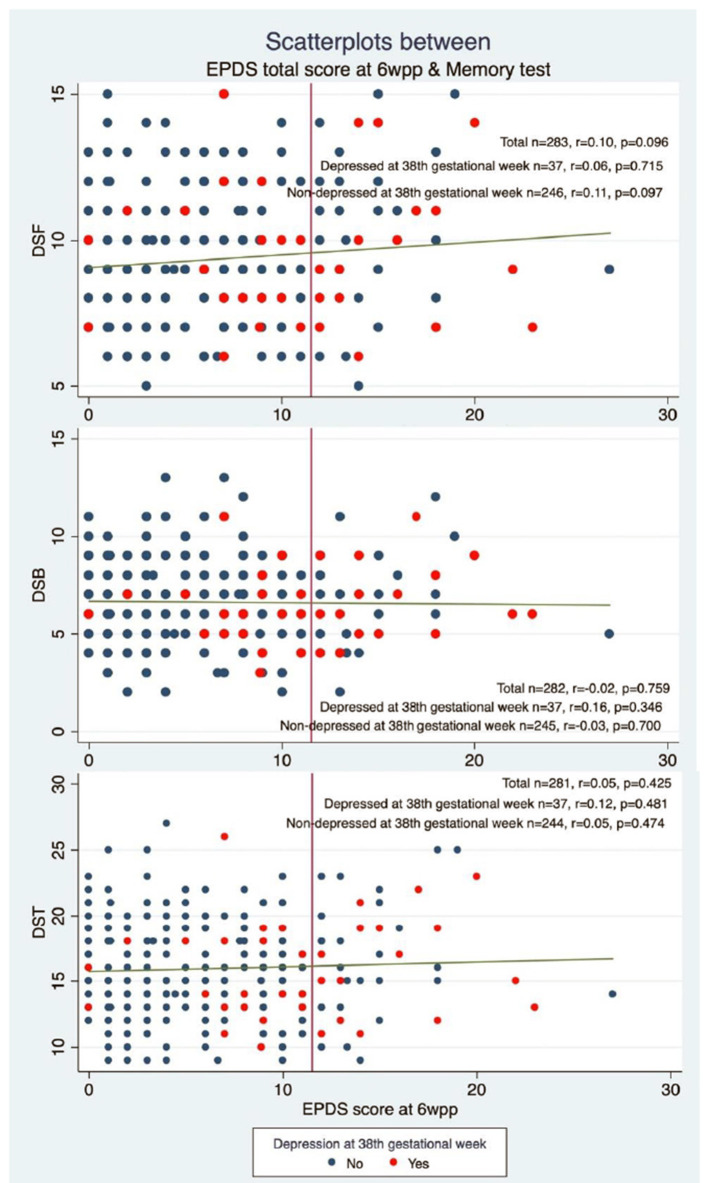
The figure presents the scatterplots showing the association between Edinburgh Postnatal Depression Scale (EPDS) (depression) score at 6 weeks postpartum (6 wpp) and memory performance on Digit Span Task stratified by having symptoms of antepartum depression or not. DSF, Digit Span Forward; DSB, Digit Span Backwards; DST, Digit Span Total which is the sum of DSF and DSB.

Spearman correlations between memory performance score and total EPDS score at 6 weeks postpartum after stratifying for depression at the 38th gestational week were not statistically significant (see Figure 2) for neither women with APD symptoms during pregnancy or those without.

## Discussion

Depressive symptoms during pregnancy were not found to be significantly associated with impairments in memory performance at the same time-point. Even though this is contrary to some previous literature featuring APD as a main predictor of short-term cognitive impairments during pregnancy (7), the finding of this study is in line with articles not finding antepartum depression to be a confounder in the association between the pregnant state and short-term cognitive impairment (1, 42). Memory impairments during pregnancy could possibly be considered primarily an outcome related to a possible different impact of pregnancy itself, and all the related and highly individualized hormonal fluctuations to different women. In fact, studies have assessed the fluctuation of hormonal levels during pregnancy and their possible relationship with memory impairments (43), showing associations with some hormones but not others (28, 44). This finding could also be attributed to methodological differences from previous studies. In fact, some of the studies showing that women with depressive symptomatology performed worse on working memory compared to their unaffected counterparts used batteries of neuropsychological tests. Since some measures are less capable of spotting individual differences, using several instruments may control for this inability; the current study, focusing on time-effective tests as possible predictors of PPD, only used the Digit Span Task.

Short-term memory in late pregnancy has been found in our study to be a predictor of PPD symptoms. The better the women's performance on the DSF in late pregnancy, the greater their risk of high depressive symptomatology postpartum. In the stratified analyses, it was the women who were not depressed antepartum or earlier in life that drove this novel finding. It does not seem that women with depressive symptoms in late pregnancy, who are at higher risk for even having depressive symptoms postpartum, were the ones performing well on the memory test either. This finding contrasts with the suggestion that individuals with lower cognitive abilities would have less cognitive flexibility and resources to manage stress-provoking conditions (19) like transitioning to motherhood. One could speculate that women with good cognitive function performing better on short-term memory during pregnancy might be high achievers in life (45, 46); for especially these women, the transition to motherhood could be an overwhelming experience, changing their life drastically, and introducing feelings of losing control which might trigger depressive symptomatology (47). Another plausible explanation that needs further exploration by using measures of autobiographical memory is that women who are not depressed during pregnancy and have better short-term memory capabilities (48) may pay more attention to, remember and might be impacted to a higher degree of negative events during delivery and afterward. Therefore, this might increase rumination and thereby the risk for depression (49, 50).

In the current study, women with a history of depression did not differ in their memory performance during late pregnancy, in contrast with the notion that previous depressive symptomatology would cause a long-lasting cognitive impairment (37). Further, women with APD did not perform worse in the memory test either. This might be due to the big inter-individual variability of how pregnancy affects cognitive abilities, possibly to a larger extend than depression. Also, theoretically, a chance of selection bias might exist; women with previous or concurrent depression but with parallelly better cognitive abilities might have chosen to participate in these visits to a higher extent. Further, both a history of, as well as APD, are some of the absolute stronger predictors of PPD (51, 52). It is thus interesting to note that the value of the memory test was mostly concentrated in predicting new-onset postpartum depressive symptomatology; this might be because women with a history of or current depression in late pregnancy are already at such high risk for PPD that the memory test has no further predictive ability.

### Strengths and Limitations

The population-based, longitudinal design and the availability of information on many possible confounding factors are major strengths of the current study. The Digit Span Task assessed as a possible predictor of PPD, is also a rather time-efficient, well-studied measure of both working memory and attention. Of note was the time of the examination because the assessment of working memory and attention occurred during late pregnancy. The singularity of this period, due to the hormonal fluctuations, the emotional unrest and the physical tiredness, complicates the results as they impact on memory performance; on the other hand, women are in very close contact with the maternity services at that time, making a possible small screening including the Digit Span Task feasible. Since the assessment took place during late pregnancy, the unique characteristics that may have influenced the memory performance, such as physical tiredness, should be taken into consideration and were adjusted for in the analyses. Relevant stratified analyses were performed, to getter better insights into the groups driving the associations.

Among the limitations is the use of a self-reporting psychometric measure instead of the more precise psychiatric interview to assess depression. Another limitation is the use of a single memory test and its one-time administration during pregnancy. Also, the relatively low acceptance rate may raise doubts about the cohort's representativeness. Baseline comparison with the general population shows participants with a sociodemographic low-risk profile, possibly due to healthy worker effect or recruitment in a highly educated university town. In general, women included in the BASIC study are a bit older, more educated and have fewer pregnancy complications in comparison to the background population, which could also affect the generalizability of the results. However, regarding the acceptance rate as such, the BASIC study may not differ substantially from other longitudinal studies in the perinatal period (53). Still, rates of clinically relevant depressive symptoms correspond to earlier reports across the whole follow-up, perhaps since loss to follow-up was relatively limited. Under-representation at baseline and higher attrition of women at risk are common challenges for prospective studies of peripartum mental health. Furthermore, the representativeness of the sub-sample may be under questioning both due to the relatively low acceptance rate and the selection process for recruitment in the sub-study. The main difference between the sub-study population and the remaining BASIC cohort is the higher rate of prior and pregnancy depression, the latest depending on the recruitment process for the sub-study, aiming at recruiting a higher proportion of depressed women. Especially among women invited to the sub-study in the research laboratory, and among depressed in late pregnancy, the ones retaining high function would have a higher possibility to attend; thereby, a degree of selection bias is to be expected and could explain the absence of association between memory performance and APD (Aim 1). Even if the results pertaining to the predictive role of memory performance for PPD (Aim 2) are driven by non-depressed women, there could still be a risk of selection bias (54) and these results should be replicated in bigger samples with better representativeness of the background population. Additionally, the “scar effect” could have better been considered in terms of the number of previous depressive episodes and their severity, instead of a yes/no variable. Finally, memory performance in the pre-conception period was not assessed; individual differences on the level of impact of pregnancy itself on memory performance might be considerable, and it is possible that pre-pregnancy memory performance could be a better predictor for both late antepartum and PPD. Future studies might take these aspects into consideration.

## Conclusion

This study provides some evidence that performance on a working memory test is not significantly associated with APD symptoms; nevertheless, it could be a predictor for new onset PPD symptoms. Among all women, but especially non-depressed during pregnancy, those scoring high on the DSF part in late pregnancy had a higher risk for PPD symptoms. Further studies will be required to further explore the pathophysiology behind, as well the predictive value of these associations.

## Data Availability Statement

The original contributions presented in the study are included in the article/Supplementary Materials, further inquiries can be directed to the corresponding author/s.

## Ethics Statement

The study has been approved by the Regional Ethical Review Board in Uppsala (Reference number 2009/171) with amendments. The patients/participants provided their written informed consent to participate in this study.

## Author Contributions

AS conceived the idea for the study together with FP and IL contributed to collection of data, supervised analyses, contributed in the interpretation of results and drafting of the manuscript. EF, IS, and EB planned and carried out the collection of data. IL worked on the data analysis and draft of the first version of the manuscript. All authors contributed to the interpretation of results, provided critical feedback comments on the manuscript, and approved the final version.

## Funding

This work was supported by the Swedish Research Council (Grant numbers 523-2014-2342, 523-2014-07605, and 521-2013-2339); the Uppsala University Hospital; the Göran Gustafsson Foundation; the Swedish Brain Foundation; Marianne and Marcus Wallenberg Foundation.

## Conflict of Interest

The authors declare that the research was conducted in the absence of any commercial or financial relationships that could be construed as a potential conflict of interest.

## Publisher's Note

All claims expressed in this article are solely those of the authors and do not necessarily represent those of their affiliated organizations, or those of the publisher, the editors and the reviewers. Any product that may be evaluated in this article, or claim that may be made by its manufacturer, is not guaranteed or endorsed by the publisher.
