# Supplementary material for: Working Memory During Late Pregnancy: Associations With Antepartum and Postpartum Depression Symptoms
Source: Front Glob Womens Health. 2022 Feb 23;3:820353. doi: 10.3389/fgwh.2022.820353 (PMC8904422; doi:10.3389/fgwh.2022.820353)
Supplement: Supplementary file 1 [file Table_1.DOCX]

| **Supplementary Table 1.** Logistic regression models evaluating the unadjusted and adjusted associations of the performance on Digit Span Task(predictor) with depression status at 6 weeks postpartum (outcome) for the women with (110) and without pre-pregnancy history of depression (n=167). | | | | | | | |
| --- | --- | --- | --- | --- | --- | --- | --- |
|  | **History of depression** | **DSF** | | **DSB** | | **DST** | |
|  |  | OR (95% CI) | p value | OR (95% CI) | p value | OR (95% CI) | p value |
| Model 1^a^ | No | 1.32 (1.04, 1.67) | **0.024** | 1.16 (0.92, 1.46) | 0.208 | 1.14 (1.00, 1.30) | **0.045** |
|  | Yes | 1.04 (0.86, 1.25) | 0.707 | 0.91 (0.71, 1.16) | 0.432 | 0.99 (0.88, 1.12) | 0.878 |
| Model 2^b^ | No | 1.28(0.99, 1.64) | 0.056 | 1.11 (0.87, 1.43) | 0.399 | 1.13 (0.98, 1.30) | 0.101 |
|  | Yes | 1.08 (0.87, 1.34) | 0.486 | 0.94 (0.73, 1.20) | 0.601 | 1.01 (0.89, 1.16) | 0.867 |
| ^a^ Unadjusted associations between the performance on the Digit Span Task and depression status at 6 weeks postpartum, based on an cut-off of 12 points on the Edinburgh Postnatal Depression Scale (EPDS)  ^b^ Adjusted associations for: education and feeling rested at assessment  DSF: Digit Span Forward, DSB: Digit Span Backward and DST: Digit Span Total which is the sum of DSF and DSB. | | | | | | | |
