# Supplementary material for: Working Memory During Late Pregnancy: Associations With Antepartum and Postpartum Depression Symptoms
Source: Front Glob Womens Health. 2022 Feb 23;3:820353. doi: 10.3389/fgwh.2022.820353 (PMC8904422; doi:10.3389/fgwh.2022.820353)
Supplement: Supplementary file 2 [file Table_2.DOCX]

| **Supplementary Table 2.** Logistic regression models evaluating the unadjusted associations of the performance on Digit Span Task (predictor) with depression status at 6 weeks postpartum (outcome) stratified by depression at the 38^th^ gestational week (n=283). | | | | | | | |
| --- | --- | --- | --- | --- | --- | --- | --- |
|  | **Antepartum Depression** | **DSF** | | **DSB** | | **DST** | |
|  |  | OR (95% CI) | p value | OR (95% CI) | p value | OR (95% CI) | p value |
| Model 1^a^ | No | 1.20 (1.01, 1.43) | **0.040** | 1.00 (0.83, 1.21) | 0.982 | 1.07 (0.97, 1.18) | 0.200 |
|  | Yes | 1.06 (0.80, 1.41) | 0.701 | 1.19 (0.83, 1.70) | 0.345 | 1.07 (0.89, 1.28) | 0.472 |
| Model 2^b^ | No | 1.25(1.03, 1.51) | **0.024** | 1.01 (0.83, 1.23) | 0.893 | 1.09 (0.98, 1.22) | 0.131 |
|  | Yes | 1.06 (0.74, 1.50) | 0.762 | 1.307 (0.86, 1.98) | 0.210 | 1.09 (0.88, 1.35) | 0.406 |
| ^a^ Unadjusted associations between the performance on the Digit Span Task and depression status at 6 weeks postpartum, based on an cut-off of 12 points on the Edinburgh Postnatal Depression Scale (EPDS)  ^b^ Adjusted associations for: education, and feeling rested at assessment  DSF: Digit Span Forward, DSB: Digit Span Backward and DST: Digit Span Total which is the sum of DSF and DSB. | | | | | | | |
